# Transcultural utility in the implementation of digital SRH interventions in sub-saharan Africa: a scoping review protocol

**DOI:** 10.3389/frhs.2026.1770265

**Published:** 2026-04-13

**Authors:** Relebohile Ntsoane, Mathildah Mpata Mokgatle, Elizabeth Nkabane-Nkholongo, B. W. Jack

**Affiliations:** 1 Department of Public Health, Sefako Makgatho Health Sciences University, Pretoria, South Africa; 2 Avedisian School of Medicine, Boston University/Boston Medical Centre, Boston, MA, United States; 3 Lesotho Boston Health Alliance, Maseru, Lesotho

**Keywords:** adolescent girls and young women, cultural utility, digital tools, intervention programs, sensitivity and relevance, sexual and reproductive health, sub-Saharan Africa

## Abstract

**Background:**

Digital health interventions (DHIs) have gained momentum in improving access to sexual and reproductive health (SRH) education and services. DHIs are increasingly recognised for reducing healthcare providers' workload, minimizing patients' long waiting times, and decreasing the distance patients must walk to access health care, thereby enhancing the quality of health services. However, the limited cultural adaptation of DHIs has undermined their usability and acceptability for improving SRH education. Evidence in sub-Saharan Africa indicates that DHIs often fall short of achieving the expected outcomes because they lack cultural relevance and are misaligned with local belief systems and sociocultural contexts. Given these gaps, this scoping review aims to systematically map existing SRH education initiatives that utilize DHIs, to assess the extent of cultural adaptation and to identify evidence-based strategies that could enhance transcultural utility in SRH DHIs.

**Methods and analysis:**

This scoping review will be guided by the framework of Arksey and O'Malley. A systematic search will be undertaken across major sources, such as PubMed, Scopus, PsycINFO, Web of Science, and other relevant sources. The review selection process will be reported using the Preferred Reporting Items for Systematic reviews and Meta-Analyses extension for Scoping Reviews (Prisma-ScR) flow diagram to ensure transparency, and EndNote will be used to eliminate duplicates during the selection of eligible studies. Eligible studies will be screened against predefined inclusion and exclusion criteria, and data will be charted to capture key characteristics, and by paying particular attention to cultural adaptation strategies of SRH-focused DHIs. Findings will be synthesised to map the current evidence base and highlight gaps for future research and practice.

**Clinical Trial Registration:**

This scoping review protocol was registered with Open Science Framework and can be accessed at https://osf.io/fx75p.

## Highlights

- This protocol explains a methodology that will be used to carry out a scoping review.
- The review focuses critically on the application of established frameworks, theories and guidelines.
- The study design involves a feasibility assessment and highlights challenges facing the implementation and sustainability of culturally sensitive SRH DHI programs.
- The results of the scoping study will highlight gaps in the body of knowledge and direct focus.
- The study will primarily focus on doing a comprehensive literature review using a scoping technique, rather than relying on evidence collected from field data, which may pose limitations on aligning theories and frameworks to achieve meaningful findings.

## 1 Introduction

Adolescent Girls and Young Women (AGYW) aged 10–24 years worldwide face several sexual and reproductive health (SRH) challenges, including unplanned pregnancies, sexually transmitted infections, and human immunodeficiency virus (HIV) infections, which pose a significant public health concern (1, 2). Furthermore, in low- and middle-income countries, AGYW have limited access to SRH services because of a lack of awareness, the judgmental attitudes of healthcare workers, and governmental and institutional limitations (3). The sub-Saharan African region accounts for 23% of the world's adolescent population aged 10–19 years. Notably, 45% of AGYW between the ages of 15 and 19 experience unintended pregnancies, which often result in unsafe abortions (4) and which account for 20% of maternal deaths as a result of pregnancy and delivery complications (5, 6).

Digital Health Interventions are technology-based solutions designed to support healthcare systems, improve health outcomes, and enhance patient engagement. They encompass a wide range of tools and platforms [mobile health (mHealth) applications, Short Message Service (SMS), interactive chatbots, Conversational agents, etc.] that leverage information and communication technologies (ICTs) to deliver, monitor, and strengthen health services. In response to AGYW SRH challenges, digital health interventions (DHIs) have gained momentum for improving access to SRH health education and services (7). DHIs are increasingly being adopted to reduce healthcare providers' workload, minimize long waiting times experienced by patients, and decrease the long distances patients have to walk to access health services, thereby enhancing the quality of health services (8, 9). However, current studies highlight the limited adaptability and sustainability of digital interventions to users' cultural contexts (10–12). Therefore, the cultural relevance and sensitivity of digital tools for SRH health education for adolescent girls and young women remain unclear in Sub-Saharan Africa.

As a result, this scoping review explores the gap in the adoption of SRH digital tools in Sub-Saharan Africa to achieve sustainable SRH interventions. Specifically, this scoping review will systematically map the evidence on the transcultural utility and cultural sensitivity of SRH DHI and program implementation strategies targeting adolescent girls and young women in sub-Saharan Africa, guided by the Population, Concept and Context (PCC) framework. The review will identify key definitions, highlight gaps, systematically map existing SRH-focused on DHIs, assess the extent of cultural adaptation, and identify evidence-based strategies to enhance cultural utility in SRH DHIs.

## 2 Methodology

**Figure 1:**
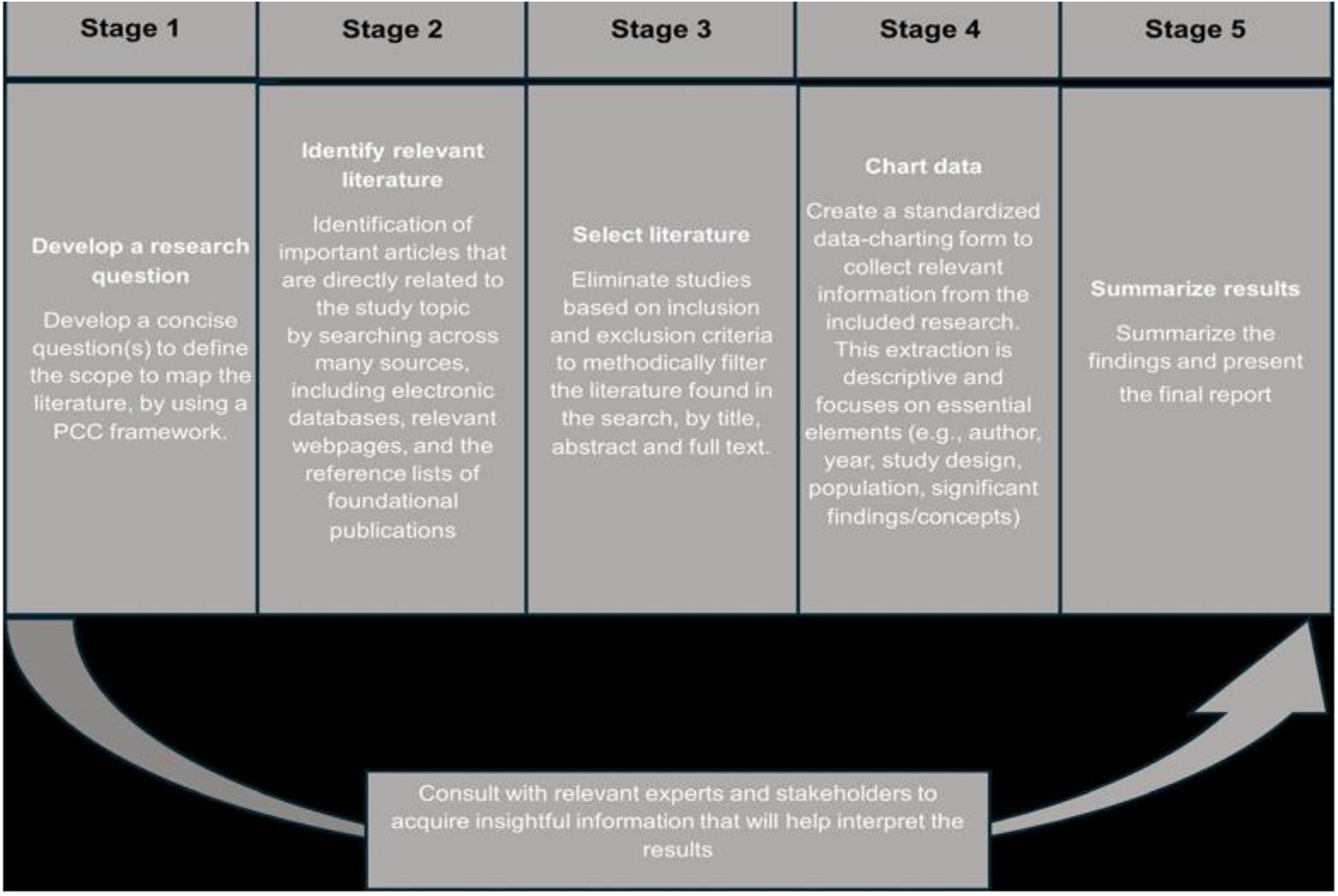
Six stages of the framework for scoping review of arksey and O’Malley (2).

This study employs a mixed-method scoping review design based on the framework developed by Arksey and O'Malley (2), which will be implemented through the following six steps: develop a research question, identify relevant literature, select literature, chart data, summarize results and consult with stakeholders. Figure 1 illustrates the thematic six-step framework for scoping reviews of Arksey and O'Malley. The Prisma-ScR flow diagram will be used to illustrate the process of the study selection.

The search strategy will involve systematic searches of articles in major electronic databases, including PubMed, Scopus, PsycINFO, and Web of Science and other relevant sources, grey literature, including Google Scholar, organizational websites such as WHO AFRO and UNAIDS, as well as specific reports issued by Ministries of Health of African countries (13). The registration of this protocol for the scoping review has been accepted by the Open Science Framework, https://osf.io/fx75p. Changes to this protocol will be reported in the results section of the final document published.

### 2.1 Stage 1: develop research questions

The PCC framework will be applied to formulate research questions with a focus on the following key elements: adolescent girls and young women (Population), transcultural utility, cultural sensitivity and relevance and SRH and digital tools (Concept) to assess the extent of cultural adaptation when SRH DHIs are implemented in sub-Saharan Africa (Context). Therefore, the following research questions will guide this scoping review:

1. What types of SRH DHIs have been implemented in sub-Saharan Africa?
2. What is the transcultural utility and significance of digital sexual and reproductive health implementation for adolescent girls and young women in sub-Saharan Africa?
3. To what extent have SRH-focused DHIs incorporated cultural adaptation strategies, and how are these strategies described in the literature?
4. What theoretical frameworks, models, or conceptual approaches have been applied to guide or evaluate the cultural adaptation of SRH DHIs?
5. What evidence exists regarding the effectiveness, usability, and sustainability of culturally adapted SRH DHIs in sub-Saharan Africa?
6. What sociocultural, linguistic or contextual factors (e.g., social norms, gender roles, traditional beliefs, language) have been identified as barriers or enablers to the implementation and access of SRH DHIs?
7. What gaps remain in the evidence base concerning cultural adaptation and the transcultural utility of SRH DHIs, and what areas warrant further investigation?

### 2.2 Stage 2: identify relevant literature

To help identify pertinent search terms and databases, we consulted an experienced librarian of the Sefako Makgatho Health Science University in South Africa. Experienced mentors in SRH and transcultural utility validated search keywords that will be used in this scoping review. The search strategies will involve a comprehensive search of major electronic databases, such as PubMed, Scopus, PsycINFO, and Web of Science. Grey literature, including Google Scholar, organizational websites such as WHO AFRO and UNAIDS, as well as specific reports from Ministries of Health in Africa, will be consulted (14). Relevant keywords will be used to identify pertinent papers through Boolean operator searches, such as “AND/OR”. The review will only consider literature published in English. Keywords will include the following components:

#### 2.2.1 Cultural component

“Transcultural” OR “Cross-cultural” OR “Cultural adaptation” OR “Cultural relevance” OR “Cultural Competence” OR “Cultural sensitivity” OR “Cultural competence” OR “Multicultural” OR “Intercultural”

AND

#### 2.2.2 Health component

“Sexual health” OR “Reproductive health” OR “Reproductive wellbeing” OR “Sexual reproductive health rights” OR “Reproductive wellbeing” OR “SRH” OR “Sexual wellbeing” OR “Sexual health services” OR “Friendly reproductive health services”

AND

#### 2.2.3 Digital intervention (strategy)

“Digital Health*” OR “eHealth*” OR “mHealth*” OR “Mobile Health*” OR “Mobile Application*” OR “Mobile Apps*” OR “App*” OR “Online Platform*” OR “Internetbased*” OR “Telehealth*” OR “Telemedicine*” OR “Digital Tool*” OR “Digital Intervention*”OR “SMS*” OR “Text Messaging*” OR “Telemedicine*” OR “Mobile Applications*” OR “Health Information Systems*” OR “eHealth*”

AND

#### 2.2.4 Public health focus strategy

“Health promotion*” OR “Health education*” OR “Health empowerment*” OR “Public health interventions*” OR “Health improvement strategies*” “Health-enhancing activity*” OR “Health awareness campaign*” OR “Behaviour changes interventions*” OR “Health supportive programmes*”.

### 2.3 Stage 3: select literature

The scoping review will be conducted using the framework developed by Arksey and O'Malley (2). This methodology will map literature according to a structured and systematic process to identify the research question and relevant studies. The reviewer will use EndNote to ensure that duplications are eliminated during the eligibility selection of relevant studies (12). Eligible studies will be determined by the PCC framework, as summarized in Table 1. This framework will guide adherence to the inclusion and exclusion criteria of the scoping review.

**Table 1:** Population, concept, context (PCC) framework.

Population (P)	Concept (C)	Context (C)
Adolescent	Transcultural utility	Sub-Saharan Africa
Girls	Cross-cultural	
Youth Teen	Cultural sensitivity	
Young woman	Cultural relevance	
	Cultural competence	
	Sexual reproductive health (SRH)	
	Reproductive wellbeing/rights	
	Sexual health services	
	Digital tools	

The selection of studies will, furthermore, follow three stages to ensure the existing evidence base is successfully mapped and research gaps are identified.

Stage 1: Screening of titles and abstracts.

Stage 2: Review of full texts of eligible articles.

Stage 3: Summarizing and data reporting through a thematic approach.

A Prisma-ScR flow diagram will be used to illustrate the process of the study selection (15).

### 2.4 Stage 4: chart data

During data charting, the reviewers will use MS Excel to include data categories, bibliographies, study programs, definitions of transcultural utility, culturally sensitive determinants of health, implementation strategies and outcomes. The PCC framework will be followed in this scoping by the selection of the following variables:

- **Population (P):** Adolescent girls and young women (AGYW) aged **10–24 years**.
- **Concept (C): Type of digital SRH tool** (Mobile Health (mHealth) Applications, Short Message Service (SMS), Social Media Platforms, Telehealth/Telemedicine, Web-based Portals, Interactive Voice Response (IVR) and Gamified Digital Tools.
- **Context (C): Sub-Saharan Africa** (All 45 countries, which are included in the WHO AFRO region and excluding Sudan, Somalia, and Mauritania).
- **Study Characteristics:** Studies **published in (2016–2026**) **and study design** (qualitative, quantitative, or mixed methods).

The detailed narrative describing the key cultural determinants, sensitivity, and SRH transcultural utility implementation strategy gaps will be identified from a thematic chart (16). The findings related to the objectives will be categorized according to the findings using a thematic approach.

### 2.5 Stage 5: summarize results

The results will be synthesised using a two-pronged approach: a descriptive numerical summary and a thematic synthesis. A descriptive numerical summary will be performed to characterize the evidence base; this data will be displayed in a table form, and figures illustrating frequency for study designs, geographic distribution among the 45 Sub-Saharan (SSA) countries will be included, along with the specific categories of digital sexual and reproductive health tools discovered. A thematic analysis will be conducted to investigate the characteristics of cultural adaptation, adhering to the framework proposed by (17). The reviewers will employ an Excel spreadsheet to organize the retrieved data, progressing through the systematic stages of familiarization, first coding, and information categorization until fundamental themes emerge.

Throughout this approach, evidence concerning the transcultural applicability and cultural sensitivity of sexual and reproductive health digital health interventions for adolescent girls and young women will be merged and mapped. The complete study selection process will be visually represented using a PRISMA-ScR flow diagram to guarantee methodological transparency.

### 2.6 Stage 6: consult with experts

Relevant stakeholders, such as academic supervisors, specialized librarians and a qualified language editor, will be consulted to achieve comprehensive dissemination of the findings. The plan for the scoping review will involve relevant stakeholders and will be part of the discussion.

## 3 Ethics

This scoping review will synthesize existing published literature and data and will not involve physical participants. Therefore, this review does not require ethics clearance from any institution. The scoping review protocol is registered through the Open Science Framework https://OSF.IO/fx75p.

## 4 Discussion and dissemination

This scoping review protocol outlines clear objectives, the rationale, methods, and design that will be applied to gather evidence on the transcultural utility of implementing SRH DHIs. This study aims to gain insight into the transcultural utility of implementing SRH programs in sub-Saharan Africa, to tailor specific SRH programs and digital health promotion tools that are culturally sensitive for adolescents, girls, and young women.

Furthermore, this paper aims to systematically map evidence on the cultural sensitivity and transcultural utility of SRH and digital health programs targeting adolescent girls and young women in sub-Saharan Africa. The review will only consider literature published in English, even though this criterion may limit the synthesis or mapping of relevant literature published in other languages. During data analysis, this scoping review will focus on recurring themes.

Ultimately, relevant stakeholders who were involved in developing the research questions will be continually consulted and engaged to ensure high-quality results. Furthermore, stakeholders will play a vital role in reviewing the scoping process to minimise bias in this review. The final findings of this review will be disseminated through publication in a recognised peer-reviewed journal and presentations at local and international conferences. Furthermore, policymakers will be engaged to enhance buy-in, effectiveness, and sustainability of culturally sensitive SRH implementation programs and culturally relevant SRH digital tools and programs for adolescent girls and young women in sub-Saharan Africa.

## References

[1] Akinwale OD Bello CB Akpor OA Elemile MG. Evaluation of adolescent/youth-friendly sexual and reproductive health services: a 7-year systematic review from January 2016 to April 2022. J Integr Nurs. (2022) 4:177. 10.4103/jin.jin_79_22

[2] Arksey H O’Malley L. Scoping studies: towards a methodological framework. Int J Soc Res Methodol. (2023) 8:19–32. 10.1080/1364557032000119616

[3] Jakobsson C Sanghavi R Nyamiobo J Maloy C Mwanzu A Venturo-Conerly K Adolescent and youth-friendly health interventions in low-income and middle-income countries: a scoping review. BMJ Glob Health. (2024) 9:e013393. 10.1136/bmjgh-2023-013393 39242132 PMC11381706

[4] Keshinro S Orah N. Improving death notification and registration: a pilot project in Lagos state, Nigeria. J Glob Health. (2024) 14:03036. 10.7189/jogh.14.03036 39421927 PMC11487468

[5] Jejaw M Tafere TZ Tiruneh MG Hagos A Teshale G Tilahun MM Three in four children aged 12−23 months missed opportunities for vaccination in sub-saharan African countries: a multilevel mixed effect analysis of demographic health and surveys 2016–2023. BMC Public Health. (2025) 25:62. 10.1186/s12889-024-21273-3 39773467 PMC11705685

[6] Danso-Appiah A Behene E Hazel JN Abanga S. Obstetric, foetal and neonatal outcomes in adolescent pregnancy in sub-saharan Africa: systematic review and meta-analysis protocol. PLoS One. (2025) 20:e0323099. 10.1371/journal.pone.0323099 40344007 PMC12063885

[7] World Health Organization. WHO guideline: Recommendations on Digital Interventions for Health System Strengthening. Geneva: World Health Organization (2019). 31162915

[8] Sanyang Y Sanyang S Ladur AN Cham M Desmond N Mgawadere F. Are facility service delivery models meeting the sexual and reproductive health needs of adolescents in sub-saharan Africa? A qualitative evidence synthesis. BMC Health Serv Res. (2025) 25:193. 10.1186/s12913-025-12344-1 39893420 PMC11786442

[9] McKinney J Kelm N Windsor B Keyser LE. Addressing health care access disparities through a public health approach to physical therapist practice. Phys Ther. (2024) 104:pzae136. 10.1093/ptj/pzae136 39288092 PMC11523610

[10] Feroz AS Ali NA Khoja A Asad A Saleem S. Using mobile phones to improve young people sexual and reproductive health in low and middle-income countries: a systematic review to identify barriers, facilitators, and range of mHealth solutions. Reprod Health. (2021) 18:9. 10.1186/s12978-020-01059-7 33453723 PMC7811742

[11] Rodgers W Murray JM Stefanidis A Degbey WY Tarba SY. An artificial intelligence algorithmic approach to ethical decision-making in human resource management processes. Hum Resour Manag Rev. (2023) 33(1):100925. 10.1016/j.hrmr.2022.100925

[12] Kaitano B. Technology and sexual health education in sub-saharan Africa. Counc Bus Soc Insights. (2023). Available online at: https://cobsinsights.org/2023/02/02/technology-and-sexual-healtheducation-in-Sub-Saharan-Africa/ (Accessed December 15, 2025).

[13] Sokhulu LH Nzimande N Makumane MA. Postgraduate students' Experiences of using EndNote in research studies. Soc Sci Educ Res Rev. (2024) 11:227–37. 10.5281/ZENODO.15258173

[14] Fromm Y Martin F Gezer T Ifenthaler D. Best practices for conducting systematic reviews: perspectives of experienced systematic review researchers in educational sciences. Technol Knowl Learn. (2025) 30:1–28. 10.1007/s10758-025-09819-9

[15] Tohit NFM Rashid SAZA Fakuradzi WFSWA Zaidi NA Haque M. Exploring pathways from community involvement to empowerment in sexual and reproductive health: a public health perspective. Adv Hum Biol. (2024) 14:296–307. 10.4103/aihb.aihb_112_24

[16] Khalil H Pollock D McInerney P Evans C Moraes EB Godfrey CM Automation tools to support undertaking scoping reviews. Res Synth Methods. (2024) 15:839–50. 10.1002/jrsm.1731 38885942

[17] Braun V Clarke V. Using thematic analysis in psychology. Qual Res Psychol. (2006) 3(2):77–101. 10.1191/1478088706qp063oa

